# Mapping and monitoring tick (Acari, Ixodida) distribution, seasonality, and host associations in the United Kingdom between 2017 and 2020

**DOI:** 10.1111/mve.12621

**Published:** 2022-10-30

**Authors:** Kayleigh M. Hansford, Sara L. Gandy, Emma L. Gillingham, Liz McGinley, Benjamin Cull, Colin Johnston, Matthew Catton, Jolyon M. Medlock

**Affiliations:** ^1^ Medical Entomology & Zoonoses Ecology UK Health Security Agency Porton Down UK

**Keywords:** citizen science, *Ixodes ricinus*, Lyme borreliosis, mapping, public health, recording

## Abstract

Tick‐borne disease risk is intrinsically linked to the distribution of tick vector species. To assess risk and anticipate disease emergence, an understanding of tick distribution, host associations, and seasonality is needed. This can be achieved, to some extent, using passive surveillance supported by engagement with the public, animal health, and public health experts. The Tick Surveillance Scheme (TSS) collects data and maps tick distribution across the United Kingdom (UK). Between 2017 and 2020, 3720 tick records were received and 39 tick species were detected. Most records were acquired in the UK, with a subset associated with recent overseas travel. The dominant UK acquired species was *Ixodes ricinus* (Ixodida: Ixodidae, Linnaeus), the main vector of Lyme borreliosis. Records peaked during May and June, highlighting a key risk period for tick bites. Other key UK species were detected, including *Dermacentor reticulatus* (Ixodida: Ixodidae, Fabricius) and *Haemaphysalis punctata* (Ixodida: Ixodidae, Canestrini & Fanzago) as well as several rarer species that may present novel tick‐borne disease risk to humans and other animals. Updated tick distribution maps highlight areas in the UK where tick exposure has occurred. There is evidence of increasing human tick exposure over time, including during the COVID‐19 pandemic, but seasonal patterns remain unchanged.

## Graphical abstract

- The risk of tick‐borne disease is linked to the distribution, seasonal activity and host associations of vector species, all of which can be informed by surveillance and citizen science
- The Tick Surveillance Scheme (established since 2005) in the United Kingdom monitors distribution changes in *Ixodes ricinus* and other native or imported vector species that may present novel health risks
- The increasing number of records received during the last 10 years and new areas of distribution for *Ixodes ricinus* suggests a possible shift in risk to public and animal health

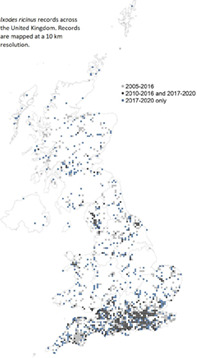

## INTRODUCTION

Ticks present an increasing risk to human and other animal health across Europe (Sprong et al., 2018). Monitoring the distribution of key vector species is important for informing tick‐borne disease epidemiology and providing an evidence‐base for public health and policy action. Surveillance, to determine distribution or abundance of vectors, is not only important for assessing the current health risk posed, but also for identifying emerging tick‐borne disease issues. This becomes ever more important at a time of significant habitat and climate change, as both can influence tick‐borne disease transmission dynamics (Medlock et al., 2013; Semenza & Paz, 2021).

Several European tick species are important from a public health perspective, including *Ixodes ricinus* (Ixodida: Ixodidae, Linnaeus), *Ixodes persulcatus* (Ixodida: Ixodidae, Schulze), *Dermacentor reticulatus* (Ixodida: Ixodidae, Fabricius), *Dermacentor marginatus* (Ixodida: Ixodidae, Sulzer), *Haemaphysalis punctata* (Ixodida: Ixodidae, Canestrini & Fanzago), and several *Hyalomma* species. Collectively, these species are responsible for the transmission of the pathogens that cause important tick‐borne diseases including Lyme borreliosis, tick‐borne encephalitis (TBE), Crimean Congo haemorrhagic fever (CCHF), anaplasmosis, babesiosis, and rickettsiosis. The changing distribution of key tick species has been reported over the last decade, along with increasing incidence of human and other animal tick‐borne diseases (Chitimia‐Dobler et al., 2019; Cull et al., 2018; Jaenson & Wilhelmsson, 2019; Medlock et al., 2018; Mierzejewska et al., 2015). Additionally, emerging pathogens have been detected and new foci of transmission reported. For example, *Borrelia miyamotoi*, which causes relapsing fever, has emerged as a human pathogen in Europe (Siński et al., 2016), and new foci have been discovered for TBE virus (Holding et al., 2019).

*Ixodes ricinus* is the most widespread tick species across Europe. It is common in most woodland habitats, but also in grazed grassland in the uplands and lowlands, and some urban habitats. Where it occurs, it is intrinsically linked to Lyme borreliosis, which has increased significantly in parts of Europe (Vandekerckhove et al., 2019), including the UK (Tulloch et al., 2020). *Ixodes hexagonus* (Ixodida: Ixodidae, Leach) is a common UK species (Abdullah et al., 2016; Cull et al., 2018) associated with hedgehogs (*Erinaceus europaeus*; Eulipotyphla: Erinaceidae, L.), cats (*Catus felis*; Carnivora: Felidiae, L.), and dogs (*Canis lupus familiaris*; Carnivora: Canidae, L.) (Jameson & Medlock, 2011), but also bites humans (Cull et al., 2018). Despite the detection of several tick‐borne pathogens in *I. hexagonus*, the significance of this species in the transmission of *Borrelia burgdorferi* sensu lato, which causes Lyme borreliosis in humans, or indeed as part of enzootic cycling of this and other tick‐borne pathogens in nature is still largely unknown (Walker, 2018).

*Dermacentor reticulatus* and *H. punctata* are two tick species of interest across Europe, largely associated with livestock and reportedly increasing or changing their distribution. *Dermacentor reticulatus* is an important vector of canine babesiosis, equine piroplasmosis, and possibly involved in the transmission of several tick‐borne viruses to humans (Földvári et al., 2016). *Haemaphysalis punctata* has been associated with several pathogens including Bhanja virus, Louping Ill virus, TBE virus, *Rickettsia*, *Borrelia*, *Babesia*, and *Anaplasma phagocytophilum* (Estrada‐Peña et al., 2017). In the UK, this species has been found infected with *Borrelia* and *Babesia* spp., has been associated with nuisance biting of humans and more recently linked to severe disease and death in lambs in the UK (Phipps et al., 2022). Although both tick species have a limited but potentially expanding distribution in the UK (Medlock et al., 2017, 2018), they have been found feeding on humans and may present a public health risk (Cull et al., 2018).

Non‐native species also are regularly detected through routine surveillance schemes. *Hyalomma* spp. have been detected outside their native range in several European countries recently, found parasitising ungulate species, but also humans in locations previously thought to be climatically unsuitable (Uiterwijk et al., 2021). *Rhipicephalus sanguineus* s.l. is perhaps not so restricted by climate, as it is able to survive and establish inside properties where pet dogs can provide bloodmeals for all life stages. Both *Hyalomma* and *Rhipicephalus* species have been detected in the UK, either on migratory birds or travelling domestic animals, and more recently, on hosts without history of travel overseas (Hansford et al., 2018; Jameson et al., 2012; McGinley et al., 2021). Detection of these species and real‐time response has likely reduced the impact on both animal and public health. For example, by alerting and providing advice and guidance to local authorities or homeowners of the potential for house infestations associated with *R. sanguineus* s.l. It also allows for rapid response to the potential incursion of Crimean‐Congo haemorrhagic fever virus, should any detected *Hyalomma* spp. test positive on follow‐up screening.

Much of the information on key tick species, particularly in the UK, has been generated through sustained surveillance, relying on submissions of ticks to a central laboratory to collate and share accurate information on distribution, seasonality, and host associations. This also includes rapid response and communication to government groups (HAIRS; Human and Animal Infections Risk Surveillance group, ACDP; Advisory Committee on Dangerous Pathogens) and other stakeholders (through local health protection teams, local authorities and veterinary publications) on possible emerging tick‐borne disease threats and the need for increased risk awareness. Passive, active, or enhanced surveillance systems are widely recognized as an integral part of preparedness and response for vector‐borne disease threats by many research and public health organizations focusing on the risk of vector‐borne diseases. The outputs of these systems are used to inform risk assessments, predictive models for tick activity and distribution, and to detect non‐native tick species or distribution changes. Longer‐term surveillance activities can provide assessments of potential changes in distribution and associated risk, as well as robust datasets for predictive modelling under future habitat or climate change scenarios.

This study reports on the last 4 years of data from the UK Tick Surveillance Scheme (TSS) which has been in place since 2005. It aims to provide up‐to‐date distribution maps for the UK of four tick species of interest (*I. ricinus*, *I. hexagonus*, *H. punctata*, and *D. reticulatus*), their hosts associations and seasonality, as well as summaries of rarer species also encountered. This is the latest instalment from a long‐term surveillance programme in the UK, which has formed the basis of similar surveillance systems in other European countries, had direct impact on public health outreach and national risk assessments on tick‐borne diseases, and provided an open channel for communication between public health officials and members of the public through citizen science.

## MATERIALS AND METHODS

The TSS relies on voluntary submissions of ticks from members of the public, veterinarians, wildlife charities, or public health specialists. Ticks are received with information on the date of tick removal; host the tick was removed from; and location information on where the tick was most likely acquired (including history of travel overseas). UK residents use a website (https://www.gov.uk/guidance/tick-surveillance-scheme#how-to-send-your-ticks-to-phe) for information on how to submit ticks. Once received, ticks are refrigerated (or frozen at −80 °C if hosts have a history of travel overseas) before being identified to species level using morphological keys (Estrada‐Peña et al., 2017; Hillyard, 1996). Ticks imported from outside of Europe and North Africa were identified using country‐ or genus‐specific keys (for example, Cooley & Kohls, 1944). Travel dates and level of engorgement were used to determine if endemic species, such as *I. ricinus*, from hosts with a history of travel overseas were likely acquired in the UK or overseas. Specimens only partially engorged and found on hosts returning to the UK very recently, for example, 1–2 days, were undetermined and not mapped. Ticks that are difficult to identify morphologically were sometimes sent to the Animal and Plant Health Agency for DNA barcoding to confirm morphological identification. A unique record is generated when a tick or multiple ticks are removed from the same host on the same date, and information is provided on the geographical location the tick was likely acquired. Each record is geocoded to the highest available spatial resolution and captured in a database for producing 10 km grid maps. Results of identification are sent to individuals submitting a tick, along with public health information on tick removal and Lyme borreliosis. Records from 2005 to 2009, and 2010 to 2016, have been published elsewhere (Cull et al., 2018; Jameson & Medlock, 2011; Pietzsch et al., 2005). Our current paper assesses all records received between 2017 and 2020, to provide an update on the latest tick surveillance data for the UK. ArcMap 10.5.1. and R (version 4.1.0) using tmap and sf packages were used to produce all maps (Pebesma, 2018; Tennekes, 2018). The package ggplot2 was used for data visualization (Wickham, 2016). Additionally, we used previously published data (2010–2016) and our most recently available surveillance data (2017–2020) to investigate how the number of *I. ricinus* records varied depending on month and host for two time periods. For each period (2010–2016 and 2017–2020), a generalized linear mixed effect model (GLMM) with a Poisson distribution was used. The response variable was the number of *I. ricinus* records and the full model included host species (humans, cats, and dogs), year, and month in its quadratic form and the interaction between host species and month as fixed variables. An observation random effect was added to account for overdispersion (Harrison, 2014). Model selection was done using the dredge function from the MuMIn package (Barton, 2020) and the model with the lowest corrected Akaike information criterion (AIC_c_) was selected. Model diagnostics were performed using the different functions available in the DHARMa package (Hartig, 2022).

## RESULTS

**FIGURE 1:**
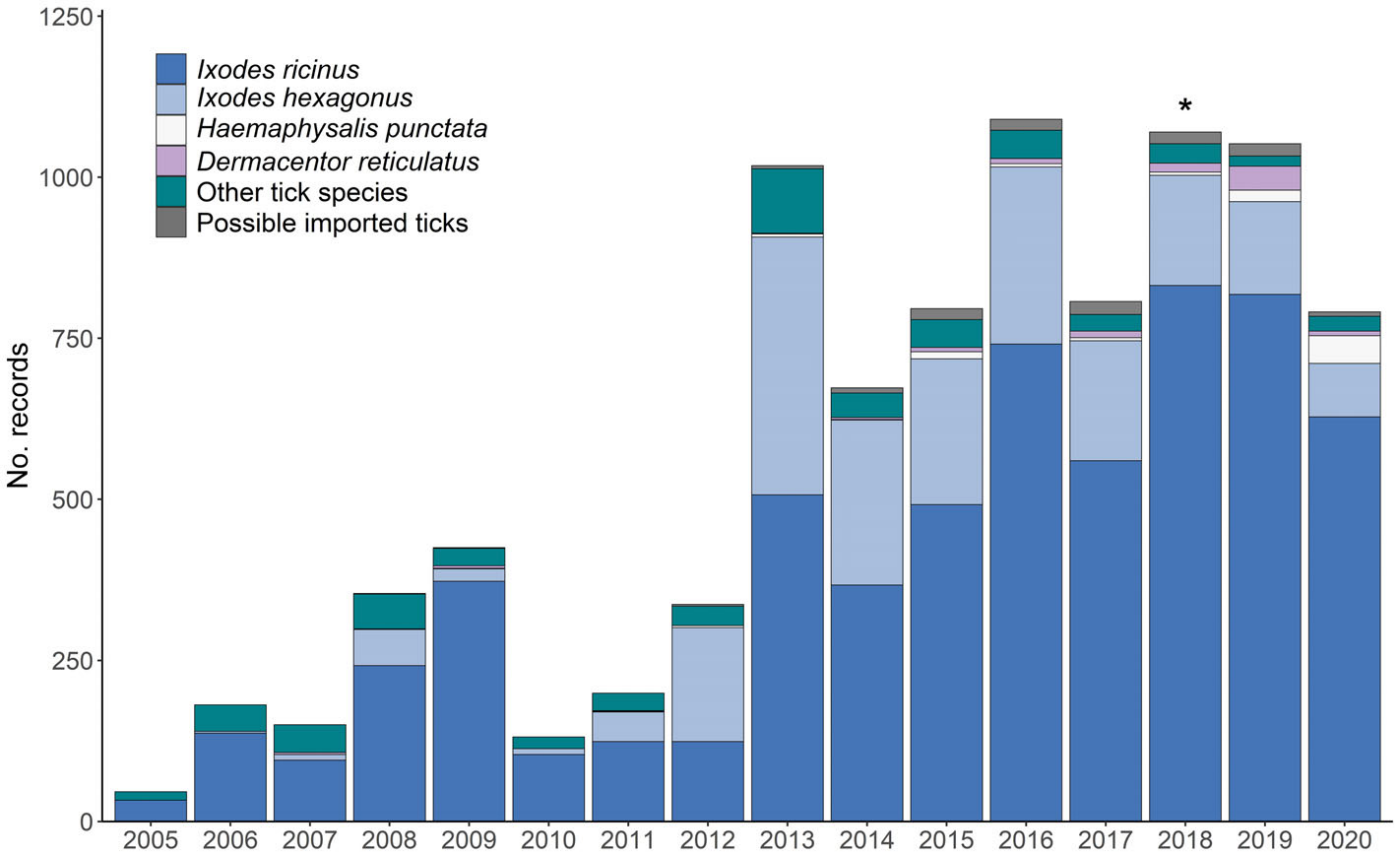
Number of records received by the UK Tick Surveillance Scheme (TSS) during 2005–2020, separated by tick species (includes data from Jameson & Medlock, 2011 and Cull et al., 2018). The * represents two records of *Hyalomma* ticks acquired in the UK.

**FIGURE 2:**
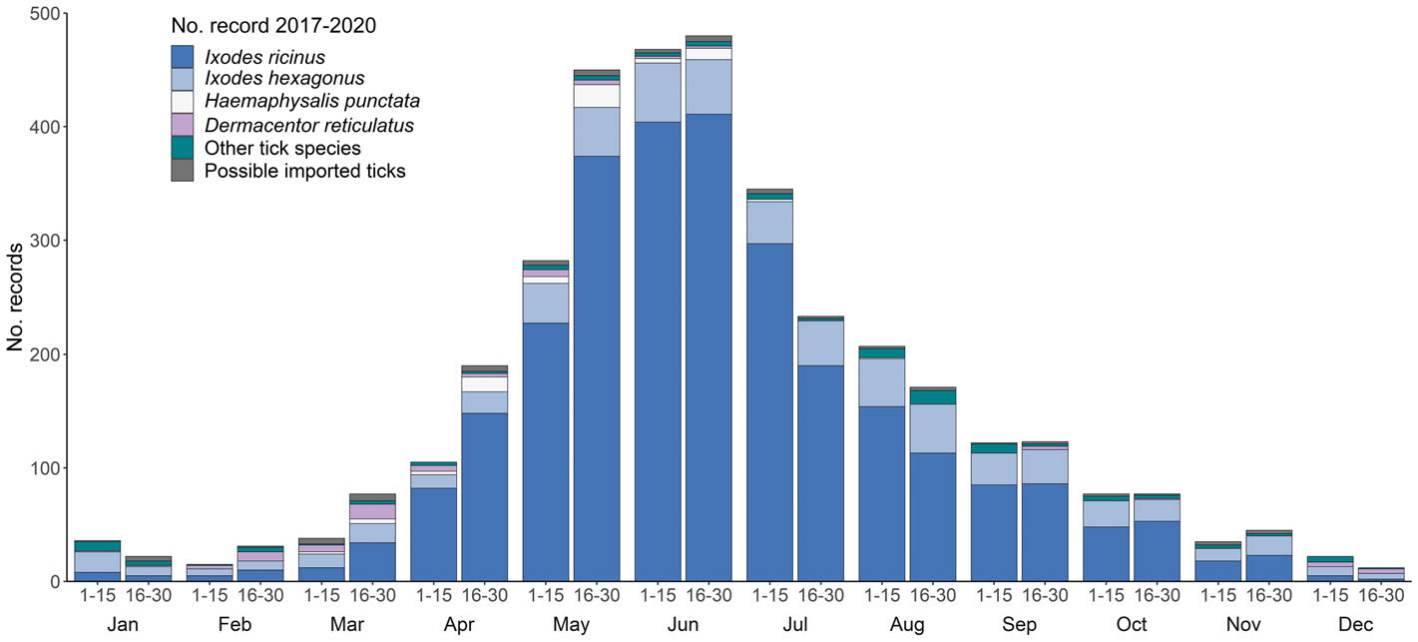
Number of records received by the UK TSS every 2 weeks per month during 2017–2020, separated by tick species.

The TSS received 3720 tick records comprised of 10,122 ticks during 2017–2020 (Table S1). The majority of tick records were associated with tick exposure in the UK (*n* = 3583; 96.3%), with 98 (2.6%) classified as imported tick records and a further 39 (1.0%) undetermined (travel dates and engorgement sizes made it impossible to determine if ticks were acquired in the UK or overseas). The total number of records received was similar in both 2017 (*n* = 771) and 2020 (*n* = 780), and in both 2018 (*n* = 1035) and 2019 (*n* = 997; Figure 1). Records were received in all months of all years (except January 2019) but numbers peaked mid‐May until the end of June for both UK acquired and records associated with a recent history of travel overseas (Figure 2).

### UK‐acquired records

Of the 3583 records acquired in the UK, there were 9590 individual ticks comprised of 5116 females (53.3%), 697 males (7.3%), 2902 nymphs (30.3%), and 875 larvae (9.1%). Percentages of life stages were similar across the years (ranging from 48.0% to 62.0% females; 5.4% to 9.0% males; 27.5% to 35.0% nymphs, and 4.1% to 14.9% larvae).

Over the 4 years, companion animals cats, dogs, ferrets (*Mustela putorius furo*; Carnivora: Mustelidae, L.), guinea pigs (*Cavia porcellus*; Rodentia: Caviidae, L.), and horses (*Equus caballus*; Perissodactyla: Equidae, L.) represented 50.0% of UK acquired records (*n* = 1790) followed closely by humans (40.1%, *n* = 1438). Ticks collected from wild mammals accounted for 5.6% of records (*n* = 200) followed by ticks collected in the environment (2.2%, *n* = 77), from livestock (1.3%, *n* = 45), wild birds (0.8%, *n* = 27), and for six records, the host was unknown (0.2%).

Thirteen tick species were identified from records acquired in the UK, including ten endemic and three non‐native species (Table S1). The dominant species was *I. ricinus* (77.5% of records, *n* = 2778 records) followed by *I. hexagonus* (16.2%, *n* = 579), *H. punctata* (2.0%, *n* = 71), *D. reticulatus* (1.8%, *n* = 65), *Ixodes frontalis* (Ixodida: Ixodidae, Panzer; 0.9%, *n* = 31), and *Ixodes canisuga* (Ixodida: Ixodidae, Johnston; 0.8%, *n* = 30). Other tick species accounted for <1% of all records and included: *Ixodes ventalloi* (Ixodida: Ixodidae, Gil Collado; *n* = 5), *Argas vespertilionis* (Ixodida: Ixodidae, Latreille; *n* = 3), *R. sanguineus* s.l. (non‐native; *n* = 3), *Ixodes trianguliceps* (Ixodida: Ixodidae, Birula; *n* = 2), *Hyalomma marginatum* (Ixodida: Ixodidae, Koch; non‐native; *n* = 1), *Hyalomma rufipes* (Ixodida: Ixodidae, Koch; non‐native; *n* = 1), and *Ixodes caledonicus* (Ixodida: Ixodidae, Nuttall; *n* = 1). Approximately, 0.4% of samples (*n* = 13) were damaged and could only be identified as *Ixodes* spp. Twenty‐three (0.6%) of all UK acquired records were found to have multiple tick species on the same host (co‐infested). Cats, dogs, and ferrets were co‐infested with *I. ricinus* and *I. hexagonus*; dogs, ferrets and foxes (*Vulpes*; Carnivora: Canidae, L.) with *I. ricinus* and *I. canisuga*; a ferret with *I. hexagonus* and *I. canisuga*; and a horse with both *H. punctata* and *I. ricinus*. The most common co‐infestation (11 records) was between *D. reticulatus* and *I. ricinus* on dogs.

### UK‐acquired host associations, seasonality, and distribution

#### Ixodes ricinus

**FIGURE 3:**
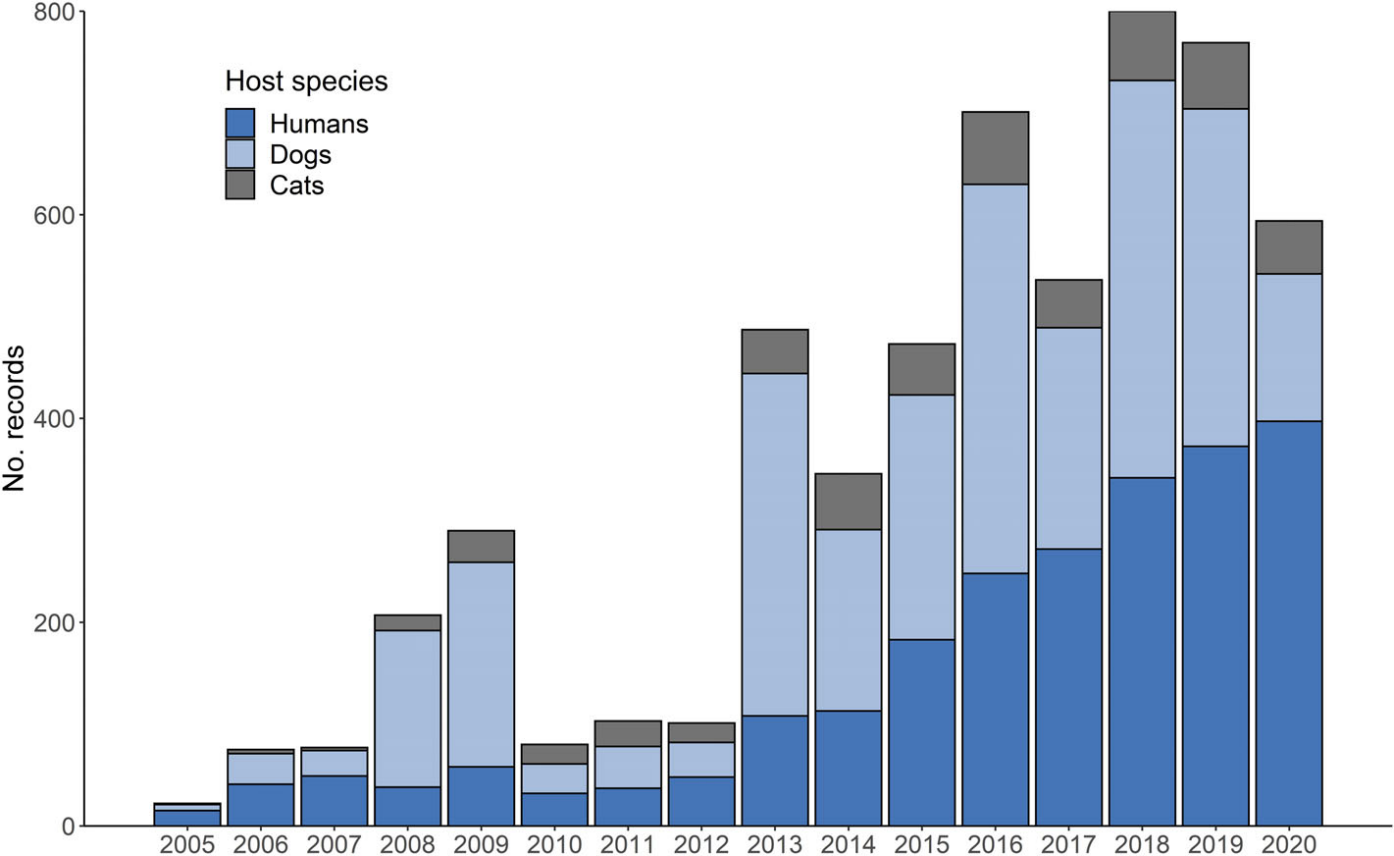
Number of records of *Ixodes ricinus* on humans (dark blue), dogs (light blue), and cats (grey) received by the TSS between 2005 and 2020.

*Ixodes ricinus* remained the most detected species through all years, with most records associated with humans (48.4%) and companion animals, particularly cats and dogs (46.7%; Table S1). The proportion of records associated with humans, dogs, and cats were similar during 2017, 2018, and 2019 (42.1%–50.8%; 40.2%–49.2%; 8.4%–9.0% respectively) but appeared to deviate during 2020 when most records were associated with humans (67.1%). The overall number of records of *I. ricinus* associated with humans also increased each year (Figure 3). *Ixodes ricinus* from wildlife were received throughout the last 4 years (Table S1), and for the first time, *I. ricinu*s was reported on three brown hares (*Lepus europaeus*; Lagomorpha: Leporidae, Pallas), a mute swan (*Cygnus olor*; Anseriforms: Anatidae), six sand lizards (*Lacerta agilis*; Squamata: Lacertidae, L.), and a siskin (*Spinus*; Passeriformes: Fringillidae). An unengorged *I. ricinus* female was also found in a vehicle. Most *I. ricinus* ticks found on humans were nymphs (68.3%), followed by females (17.0%), larvae (12.6%), and males (2.1%; Table S1). By contrast for companion animals, female‐stage ticks made up the overwhelming majority (81.6%) compared to males or juvenile life stages (15.3% and 3.1% respectively; Table S1).

**FIGURE 4:**
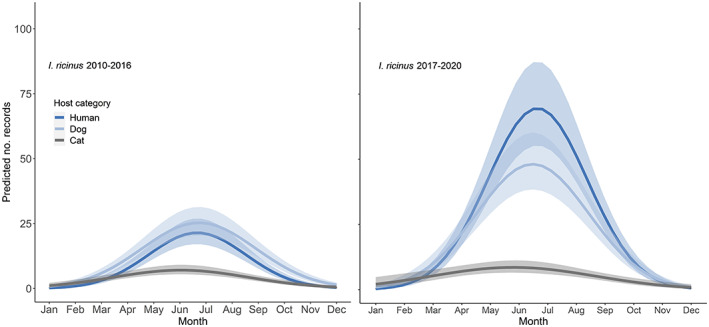
Predicted number of *Ixodes ricinus* records on humans (dark blue), dogs (light blue), and cats (grey) in 2010–2016 and 2017–2020. Solid lines represent the predicted number of records. Shaded areas represent 95% confidence intervals.

*Ixodes ricinus* acquired in the UK were recorded during all months of the year, including during the winter months of December to February when 35 records comprised of 59 ticks (all life stages except larvae) were reported. The number of records started to increase during spring (March–May, *n* = 874) and summer months (June–August, *n* = 1557), peaking in June (*n* = 812), and then falling steadily during autumn (September–November, *n* = 312). The selected models to explain how records of *I. ricinus* varied depending on year, season, and host included the interaction between host species and month (Tables S2 and S3). This interaction between host species and month was significant in the models for both 2010–2016 (*χ* ^2^[4] = 29.05, *p* < 0.001) and 2017–2020 (*χ* ^2^[4] = 20.36, *p* < 0.001). The predicted number of records during 2010–2016 and 2017–2020 followed the same seasonal pattern, with overall predicted record numbers increasing before reaching a peak in June–August and human records making up the majority in recent years, followed by dogs and cats (Figure 4). Of note is the consistent predicted peak in *I. ricinus* found on cats (early June) which precedes the predicted peak on dogs and humans (late June).

**FIGURE 5:**
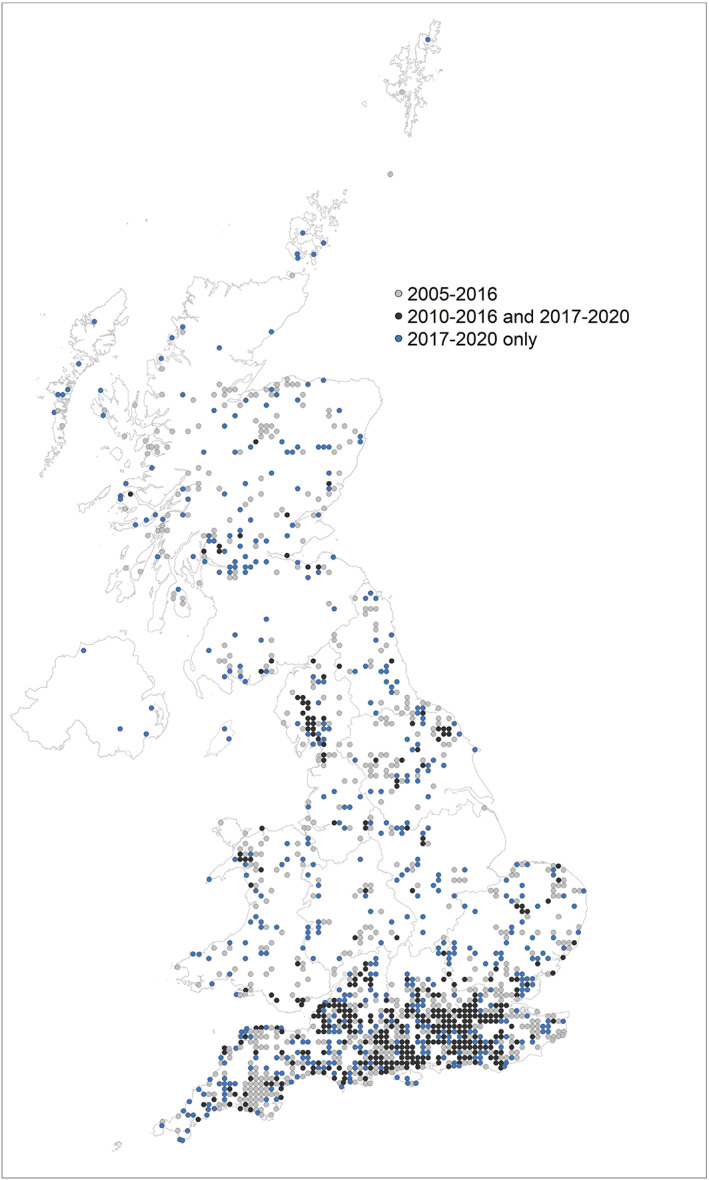
Distribution of records of *Ixodes ricinus* in the UK mapped at 10 km resolution. Grids with *I. ricinus* presence reported between 2005 and 2016 only are light grey. Those in dark grey had records during 2010–2016, and 2017–2020 and represent persistent reporting of this species in these areas. Grids in blue have records of *I. ricinus* reported for the first time during 2017–2020 and may indicate new areas of distribution. Contains Ordnance Survey data © Crown copyright and database right 2022. Contains National Statistics data © Crown copyright and database right 2022.

The distribution of *I. ricinus* continues to span the whole of the UK, as far north as the Shetland Islands in Scotland and as far south as the Lizard peninsula in Cornwall, England. Fewer records were reported from Wales (Figure 5), and only four from Northern Ireland. Records are still primarily being reported from Southern England, where persistent reporting has occurred in several regions since 2010, along with records being reported in new 10 km grids for the first time during the past 4 years (Figure 5). Cumbria, North Yorkshire, and Snowdonia also show persistent reporting and new 10 km grids with records for the first time (Figure 5). Very few records were received from the East Midlands.

#### Ixodes hexagonus

**FIGURE 6:**
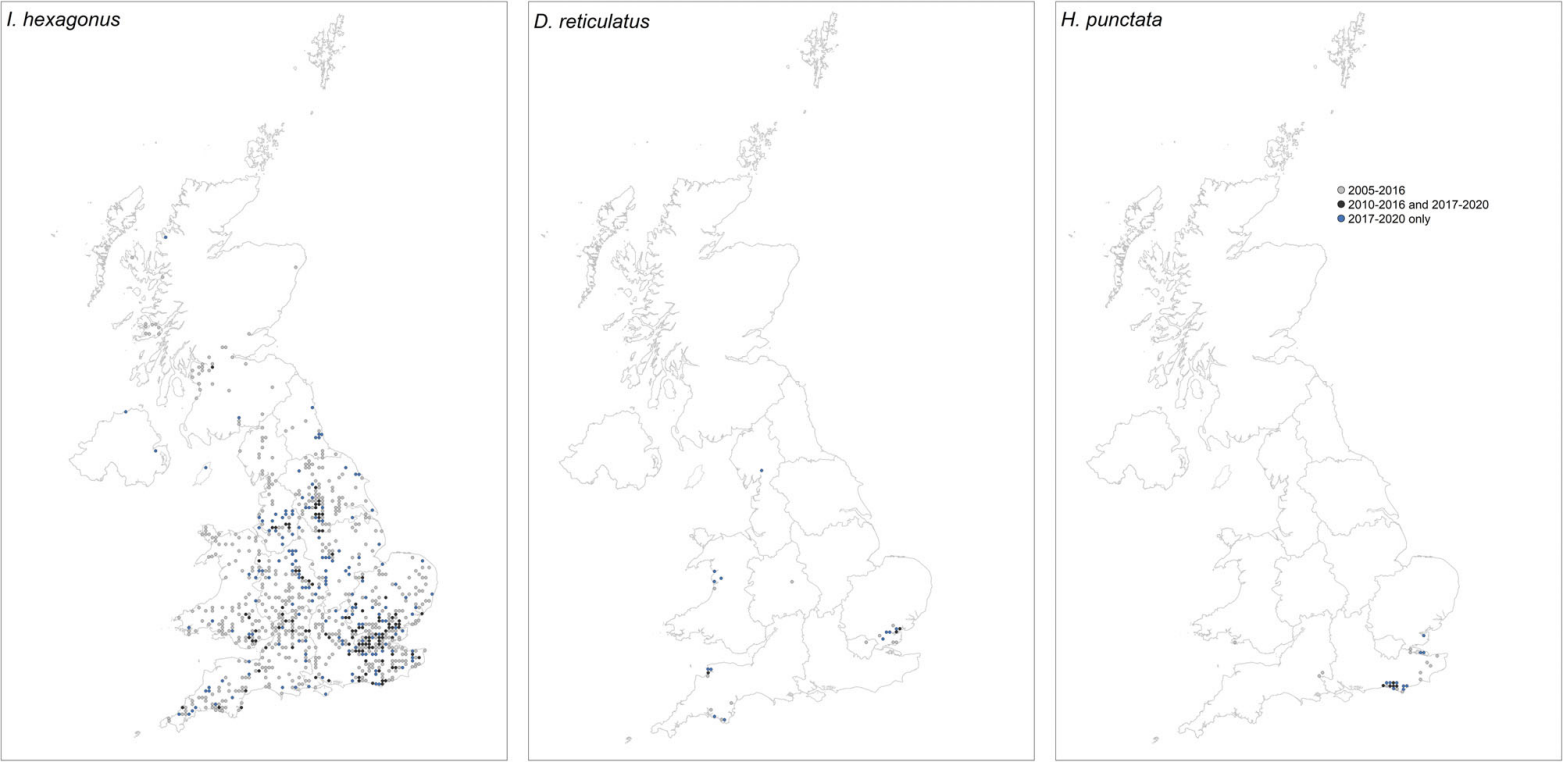
Distribution of records of *Ixodes hexagonus*, *Dermacentor reticulatus*, and *Haemaphysalis punctata* in the UK mapped at 10 km resolution. Grids with presence reported between 2005 and 2016 only are light grey. Those in dark grey had records during 2010–2016, and 2017–2020, and represent persistent reporting of this species in these areas. Those in blue have records reported for the first time during 2017–2020 and may indicate new areas of distribution. Contains Ordnance Survey data © Crown copyright and database right 2022. Contains National Statistics data © Crown copyright and database right 2022.

The second most common tick species detected in UK acquired records was *I. hexagonus*. Of the 579 records (Table S1), the majority were associated with dogs (37.0%), followed by cats (28.2%) and hedgehogs (19.1%). Forty records (6.9%) were associated with humans, and the remaining records were associated with foxes (3.8%), ferrets (1.2%), badgers (*Meles meles*; Carnivora: Mustelidae, L., 0.5%), and sheep (*Ovis aries*; Artiodactyla: Bovidae, L., 0.3%). Single records were captured from a horse, pig (*Sus scrofa*; Artiodactyla: Suidae, L.), and polecat (*Mustela putorius*; Carnivora: Mustelidae, L) and five records were found indoors (likely from pet cats or dogs). Finally, six records were found in gardens. Eight of the human *I. hexagonus* records were associated with tick exposure in private gardens, and three specifically mentioned the presence of hedgehogs/feeding stations. Female *I. hexagonus* were the most common life stage identified (50.2%), followed by nymphs (34.0%), larvae (13.8%), and males (2.0%; Table S1). This trend was generally the same for all host species, except for dogs, where the proportion of larvae (40.1%) was higher compared to all other life stages (nymphs 34.3%, females 24.8%, and males 0.8%). Record numbers decreased per year from 186 during 2017, to just 83 during 2020 (Figure 1). Records of *I. hexagonus* were reported every month of the year, with a higher proportion of records captured May–June (Figure 2). All records associated with humans were acquired in the UK, with around 10 records captured per year between the months of February and November. Most records associated with humans were blood‐fed ticks, with just three records of ticks crawling on clothing and possibly associated with contact with pet cats or dogs. Most records were reported from England, with fewer in Wales. *Ixodes hexagonus* was rarely reported in Scotland or Northern Ireland (Figure 6).

#### Haemaphysalis punctata

Records of *H. punctata* (*n* = 71) were mostly on horses (53.5%) and humans (33.8%). Most ticks were attached and feeding when found. Other records included *H. punctata* on sheep (5.6%), dogs (5.6%), and a cat (one male tick found crawling). Record numbers increased considerably each year, from five in 2017 to 43 in 2020 (Figure 1), with some reports of ticks acquired in a town park. Records were received January–June, with a peak observed during May each year (Figure 2). All records were reported from South East England, specifically Essex (Maylandsea, Dengie Peninsula), Kent (Isle of Sheppey), East Sussex, and West Sussex (South Downs National Park, Brighton, Eastbourne, and Lewes) (Figure 6).

#### Dermacentor reticulatus

Records of *D. reticulatus* acquired in the UK (*n* = 65) were primarily associated with dogs (69.2%) or found indoors likely having fed on dogs (9.2%; Table S1). The remaining *D. reticulatus* records were associated with ticks crawling on or feeding on humans (21.5%; Table S1). The highest number of records of this species were recorded during 2019, and this was associated with an increased number of records from North Devon. Records were received January–June, during September, October, and December, with the majority reported during March (Figure 2). Most records were reported from known locations in North Devon (Braunton Burrows), South Devon (near Plymouth), Essex (Danbury, Great Wakering, Ramsden Heath, and Tollesbury), and Wales (Aberystwyth, Aberdovey, and Fairbourne) (Figure 6). Most records in North and South Devon were associated with dogs (*n* = 33), with two records from humans, although neither were attached and feeding. In Essex and Wales, the proportion of records from dogs and humans was different, with human records appearing to be more common. This included six records of *D. reticulatus* found crawling on humans, and five records of female *D. reticulatus* feeding on humans. Away from established populations, a record also was reported from a dog in Southeast Cumbria, in June 2018. The dog had no history of travel to any other known locations where *D. reticulatus* are established.

### Less common tick species acquired in the UK

*Ixodes frontalis* was identified from 32 records all acquired in the UK. Records were primarily associated with wild birds (35.2%; Table S1). Additionally, five (7.0%) records were associated with tick bites on humans, including one that mentioned possible tick exposure in a private garden. All ticks found on humans were female and attached and feeding on the head or face. Two fully engorged females also were found in gardens. *Ixodes frontalis* were found on birds between April and December, but the majority (50.0%, *n* = 16) were from August. At least one record from a human was reported each year, with four of the five records associated with ticks removed between August and October (the other record was from May). Most records were reported from central and southern England with several also from Wales.

All 30 records of *Ixodes canisuga* were UK acquired and most associated with foxes (75.9%). Other records were from dogs, ferrets, and a badger (Table S1). Most ticks found were females (64.5%), followed by larvae (21.5%) and nymphs (14.0%). Like *I. frontalis*, no males were submitted to the TSS. Records were reported during almost all months of the year (except August and October), but most were associated with January and December. Records were mostly reported in England, with fewer records in Wales and Scotland.

The remaining UK‐acquired records (*n* = 14) were associated with tick species rarely reported (Table S1). These included six records of *I. ventalloi* from companion animals (cats and dogs), three records of *A. vespertilionis* associated with bat roosts in properties, two records of *I. trianguliceps* (associated with dogs) and one *I. caledonicus* from a chough (*Pyrrhocorax pyrrhocorax*; Passeriformes: Corvidae). Additionally, two UK‐acquired *Hyalomma* species were detected for the first time in England. A male *Hy. marginatum* was found crawling on a human in Norfolk, and a male *Hyalomma rufipes* was attached to a horse in Dorset, both cases of which have been reported elsewhere (Hansford et al., 2019; McGinley et al., 2021). Finally, three records of *R. sanguineus* s.l. were reported on dogs with no history of travel outside the UK. Two records were associated with travel by the dog owners to endemic countries, where they may have inadvertently transported this non‐native tick into the UK in luggage. For the third case, the source of the ticks could not be established, but resulted in a significant number of ticks (>70 reported by the recorder) being found inside the home (as well as one in the garden).

### Records with a history of travel overseas

**FIGURE 7:**
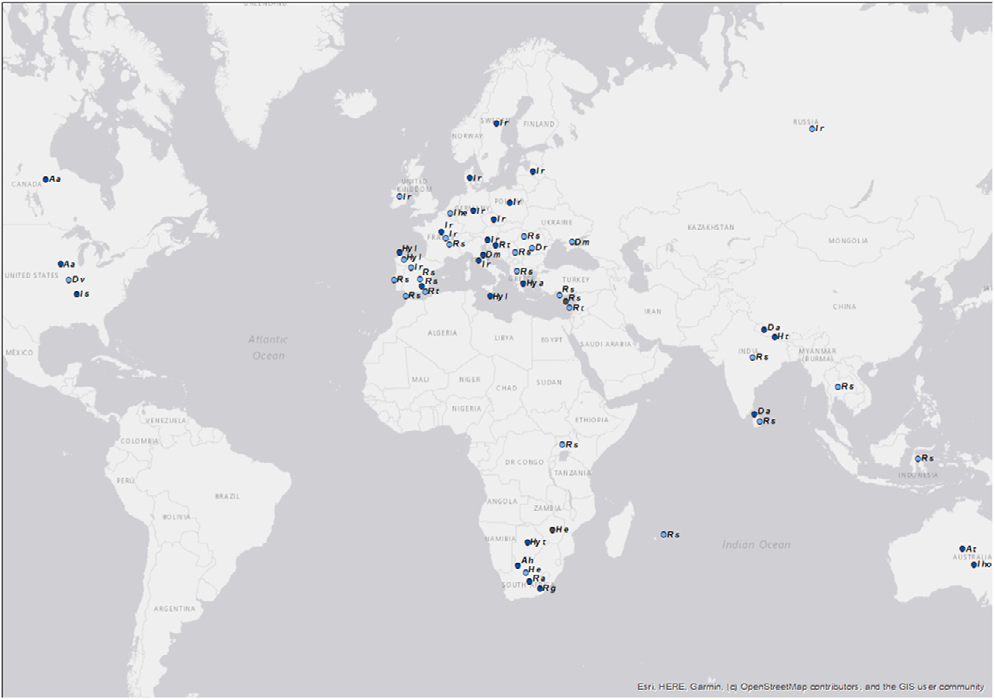
Origin of ticks imported into the UK on travelling cats (grey), dogs (light blue), and humans (dark blue) reported to the TSS between 2017 and 2020 (includes data from (Gillingham et al., 2020; Hansford et al., 2018). Dots on the map may represent one or more records and are not the exact locations where ticks were acquired (they are at country centroids where possible). *A a*, *Amblyomma Americanum*; *A h*, *Amblyomma hebraeum*; *A t*, *Amblyomma triguttatum*; *D a*, *Dermacentor auratus*; *D m*, *Dermacentor marginatus*; *D r*, *Dermacentor reticulatus*; *D v*, *Dermacentor variabilis*; *H e*, *Haemaphysalis elliptica*; *H t*, *Haemaphysalis tibetensis*; *Hy a*, *Hyalomma aegyptium*; *Hy l*, *Hyalomma lusitanicum*; *Hy t*, *Hyalomma truncatum*; *I he*, *Ixodes hexagonus*; *I ho*, *Ixodes holocyclus*; *I r*, *Ixodes ricinus*; *I s*, *Ixodes scapularis*; *R a*, *Rhipicephalus appendiculatus*; *R g*, *Rhipicephalus gertrudae*; *R s*, *Rhipicephalus sanguineus* s.l.; *R t*, *Rhipicephalus turanicus*. This map was produced using ArcMap 10.5.1 and contains data from Esri, HERE, Garmin, © OpenStreetMap contributors, and the GIS User Community.

A total of 137 records were associated with ticks on hosts with a recent history of travel overseas, the majority consisting of ticks removed from dogs (51.8%) and humans (43.8%). Ninety‐eight records were determined to be imported and included 20 different tick species from 36 countries (Table S1; Figure 7).

*Rhipicephalus sanguineus* s.l. accounted for most imported tick records (36.7%, Table S1). All but one record of *R. sanguineus* s.l. was from dogs, most of which were recently imported from Spain (*n* = 10), Greece (*n* = 5), Romania (*n* = 5), Cyprus (*n* = 4), and Indonesia (*n* = 2). All other countries (Figure 7) had single records associated with imported dogs. A record of *R. sanguineus* s.l. was imported on a child returning from a trip to Spain. The second most common species of imported tick records was *I. ricinus* (29.6%, Table S1), with the majority associated with history of travel to France (*n* = 9), Germany (*n* = 3) and Italy (*n* = 3). *Ixodes ricinus* importations were mostly associated with humans (65.5%), with the remaining records associated with dogs (34.5%, Table S1). All other countries had just one or two records associated with recently travelled humans or dogs (Figure 7, Table S1), and for all other tick species, there were only four or fewer records associated with a history of travel overseas (Table S1; Figure 7).

## DISCUSSION

During the first 8 years of surveillance, the TSS managed a small number of submissions per year (Jameson & Medlock, 2011) but provided significant updates to historical tick distribution maps (Pietzsch et al., 2005). Since 2013, the TSS has played a more important role informing UK public and animal health on the distribution of common species, and occurrence and emergence of rarer and imported species (Cull et al., 2018). The TSS now consistently collates over 1000 records each year and can be considered a sentinel indicator for imported ticks, either from human travellers, travelling or imported pets, or from migratory birds (Gillingham et al., 2020; Hansford et al., 2018; McGinley et al., 2021). This demonstrates the wealth of data that can be achieved using passive surveillance initiatives and has the added benefit of engagement with members of the public.

Since 2013, the numbers of records received has steadily increased, particularly during the last 4 years, and with this, an increasing range in distribution of the most common species, *I. ricinus*, has likely occurred (Cull et al., 2018). Other key findings during the last 4 years include an increase in records of ticks removed from humans, including a shift in the overall proportion of human records compared to other host groups, notable increases in the number of submissions of both *D. reticulatus* and *H. punctata*, a possible increase in the exposure of humans to *I. hexagonus* and *I. frontalis*, and the increasing exposure to non‐native ticks amongst hosts without a history of travel overseas.

Increasing submissions to the TSS over the last 8–9 years may be due to changing tick distributions, which have been reported for many tick species across Europe (Chitimia‐Dobler et al., 2019; Cull et al., 2018; Jaenson & Wilhelmsson, 2019; Medlock et al., 2018; Mierzejewska et al., 2015). Increasing awareness of ticks more generally, supported by the impact of high‐profile Lyme borreliosis cases reported in the media, and local awareness campaigns should not be underestimated, however, and may also have contributed to increased submissions. The numbers of records from humans have consistently risen every year since 2010, with the remaining submissions varying from 1 year to the next, particularly for dogs. Indeed, 2020 saw a marked decline in submissions of ticks removed from dogs, possibly owing to fewer visits to veterinarians during the COVID‐19 pandemic. Some veterinary practices were only able to offer emergency appointments during this time, meaning there would have been less opportunity to engage with the TSS. Records of ticks removed from humans during the same year were the highest so far reported to the scheme, and similar increases in reports of tick bites were recorded elsewhere in Europe during this time (Borșan et al., 2021). Records from humans accounted for 67% of all records received in 2020; up from a 40% to 50% average between 2017 and 2019. This is a marked increase on previous years (24% Jameson & Medlock, 2011; 31% Cull et al., 2018). This may reflect increased outdoor activity during the pandemic, following a period of restricted access to outdoor spaces, and subsequent exposure to tick bites. This could have included individuals already aware of the risks associated with ticks, but would also likely have included those who might not normally use local outdoor areas and lack tick awareness. Whether this has resulted in increased Lyme borreliosis cases in the UK remains to be determined. One study in Poland investigated the potential impact of the pandemic on Lyme borreliosis incidence (Piotrowski & Rymaszewska, 2022). A significantly lower incidence compared to annual rates in previous years was reported, but the study authors expressed serious concern over the potential lack of visits to health care providers during the pandemic that may have resulted in cases being untreated.

Over 96% of tick submissions to the TSS are acquired in the UK and the majority are made up of the key vector species, *I. ricinus*. The hazard posed by ticks acquired in the four nations (England, Wales, Scotland, and Northern Ireland) remains a concern, particularly for transmission of Lyme borreliosis. Additionally, the recent detection of TBE virus in *I. ricinus* and probable autochthonous cases acquired in England (Holding et al., 2019; Mansbridge et al., 2022), the recent near fatal human case of bovine babesiosis in England (Johnson et al., 2020), and the potential risk of acquiring other tick‐borne pathogens such as *Anaplasma phagocytophilum* (Gandy et al., 2022), *Borrelia miyamotoi* (Hansford et al., 2015), and *Rickettsia* sp. (Tijsse‐Klasen et al., 2013) highlight the importance of continued surveillance and research on tick‐borne diseases.

A range of UK tick species exhibit a distinct seasonality, which could shift with a changing climate (Medlock et al., 2013). *Ixodes ricinus* and *I. hexagonus* were submitted to the TSS all year round, with a distinct reduction in submissions during the winter months of December–February. The main season for submissions starts to increase in late April, peaking in late May–late June, with a marked reduction in August. There is a little evidence of a second peak of tick submissions in the autumn. This pattern remains unchanged from previous years (Cull et al., 2018; Jameson & Medlock, 2011), suggesting that climate effects are not yet causing significant shifts in the pattern of *I. ricinus* activity in the UK, and the highest risk periods for tick bites continue to be spring and early summer. What is noticeable is the persistent earlier peak in submissions of *I. ricinus* from cats than from dogs and humans (Cull et al., 2018). This has been reported before when analysing data from veterinary practices and could potentially be used as an early indication of subsequent tick exposure for dogs and humans (Tulloch et al., 2017).

During the last 4 years, the TSS largely received ticks removed from companion animals (50%) and humans (40%), and although there are fewer records of ticks removed from wildlife compared to previous years (Cull et al., 2018), the species host range was varied. The TSS does not get many submissions from livestock (1.3%) and thus has limited value for detecting livestock‐associated tick issues. The predominant tick stage recorded remains the nymphal stage for humans (~65%) and adult females on companion animals (~72%) (Cull et al., 2018; Jameson & Medlock, 2011). Approximately, 77% of tick submissions during the last 4 years were *I. ricinus*, which is similar to that reported by Jameson and Medlock (2011) (71%), but higher than Cull et al. (2018) (~59%) which reported a higher proportion of *I. hexagonus*. The higher numbers of *I. hexagonus* records reported previously were likely due to the greater proportion of ticks removed from wildlife.

*Ixodes ricinus* continues to be commonly reported in southern England counties, but there are many new records around the English midland counties, as well as in adjacent, pre‐existing endemic areas of the Lake District, North Yorkshire Moors, East Anglia, and Snowdonia. New areas with records of *I. ricinus* in mid‐Wales and the Welsh marches are notable, because historically, *I. ricinus* has not been reported to the TSS from these areas. Comparisons between TSS data and Lyme borreliosis incidence data would be useful to determine if new potential areas of *I. ricinus* spread can be linked to clinical disease outcomes and both could potentially justify increased investment in tick awareness measures at a local level. Tick submissions for Northern Ireland, Wales, and Scotland are likely to be under‐reported, and this may be due to the lower incidence of Lyme borreliosis reported in Wales, but for Scotland and Northern Ireland, it is likely that there have been limited submissions to the Public Health England (now UK Health Security Agency) Scheme.

This paper provides an updated map of *I. hexagonus* in the UK, providing additional data to those published in recent years (Abdullah et al., 2016). This species continues to be the second most reported in the UK, with a shift in submissions towards infestations on companion animals compared to wildlife during the last 4 years, likely driven by a reduction in submissions of ticks removed from wildlife hosts. Records occur widely across England and parts of Wales, with notable areas around London and the south‐east, Gloucestershire, the West Midlands, and West Yorkshire. These often coincide with large urban conurbations where companion animals are likely exposed to urban *I. hexagonus* populations, and usually in areas where *I. ricinus* records are less common. Although known as the hedgehog tick, this species is not confined to this host, with records more commonly associated with dogs and cats, but also with several other wildlife species. However, *I. hexagonus* is known to heavily infest hedgehogs (Walker, 2018), and can often be reported in urban gardens where some human tick bites have been reported. Although far less common than *I. ricinus*, over the last 4 years there have been 40 records of *I. hexagonus* on humans, far greater than reported previously. The peak in submissions, however, is the same as *I. ricinus*, meaning that current guidance on tick avoidance during peak activity periods remains relevant for both key species.

Other tick surveillance papers have specifically reported on *H. punctata* in areas of Sussex, Kent, and Essex (Medlock et al., 2018) and *D. reticulatus* in Wales, Devon, and Essex (Medlock et al., 2017), and distributions reported during the last 4 years continue to provide evidence of established populations in these areas. Additionally, further evidence of human tick bites from both species is provided, and the frequency appears to be increasing. *Ixodes frontalis* records were primarily from birds, but five records of biting on humans are an increase on previous reports of two in Cull et al. (2018). Most of these records were received August through October, at a time of year when *I. ricinus* records are much reduced. The public health significance of human tick bites from this species, along with *H. punctata* and *D. reticulatus* require further investigation. Many of the other species, except perhaps *I. canisuga* mainly found on foxes, are quite rare submissions to the TSS. These records are usually from individuals who specifically work with bats, seabirds, or small mammals.

UK‐acquired non‐native species of the genera *Hyalomma* and *Rhipicephalus* are perhaps a portent for the future, not just for the UK but indeed elsewhere in Europe where similar occurrences have been reported (Uiterwijk et al., 2021). *Hyalomma* are imported into the UK on migratory birds, and the accounts of UK‐acquired records have been reported elsewhere (Hansford et al., 2019; McGinley et al., 2021), as have reports of *R. sanguineus* s.l. on dogs following travel (Hansford et al., 2018). It is of particular interest, however, that three records of *R. sanguineus* s.l. were detected on dogs with no history of travel. All three were likely exposed to ticks imported from other hosts with history of travel and highlights the importance of gaining a full history of possible exposures when investigating similar instances. It will be of public and animal health importance to monitor submissions of these ticks into the future.

The TSS often receives records of ticks acquired overseas (Gillingham et al., 2020; Hansford et al., 2018), which can present an additional challenge for public health. The proportion of imported records remains unchanged compared to previous years, however, and they continue to be dominated by *R. sanguineus* s.l. on travelling dogs and *I. ricinus* on humans. The additional six new species reported on travellers in the UK since 2016, and all imported records spanning five continents and 36 countries highlights the opportunity for ticks to move great distances, and potentially present novel public or animal health threats to individuals bitten. Although imported human tick‐borne diseases are uncommon in the UK, around 15% of Lyme borreliosis cases diagnosed in the UK are associated with travel overseas. It is imperative to encourage tick treatments for travelling or imported pets, and to highlight to all prospective travellers of the risk of ticks acquired overseas.

The TSS has now been running for 16 years, and continues to synthesize information on species distributions, host associations, and activity periods, as well as enabling real‐time response to emerging tick and tick‐borne disease related threats. Assessments of changes over time and widespread engagement at the local level, and with the public, on tick awareness and related issues are other important outputs. Although our tick distribution maps will not fully reflect the distribution of Lyme borreliosis in the UK, they do provide an indication of where tick exposure may occur. The public health impact of the TSS and similar schemes (e.g. Jongejan et al., 2019) should not be underestimated. Other countries could benefit from engagement with passive surveillance or programmes such as VectorNet (ECDC, 2019), particularly with the changing distribution of key vector species and their associated disease risks.

## AUTHOR CONTRIBUTIONS

Kayleigh M. Hansford manages the TSS and coordinated the development of this publication with Jolyon M. Medlock. Both were also involved in data analysis, interpretation, drafting, and revising the manuscript. Sara L. Gandy was involved in data acquisition, analysis, as well as drafting and revising the manuscript. Emma L. Gillingham, Liz McGinley, Benjamin Cull, Colin Johnston, and Matthew Catton were involved in data acquisition and revision of the manuscript. All authors approved the final version of the manuscript submitted.

## Supporting information


**Table S1:** Tick Surveillance Scheme records received 2017–2020, detailing species, host associations [number of records per host species/environment], number of records, number of larvae (L), nymphs (N), adult females (F), adult males (M), and total ticks received.

**Table S2:** Outputs from the generalized linear mixed effect model (GLMM) explaining the effects month, host, and year on the number of *I. ricinus* records submitted through the TSS in 2010–2016
**Table S3:** Outputs from the GLMM explaining the effects month, host, and year on the number of *I. ricinus* records submitted through the TSS in 2017–2020

## Data Availability

Data from the Tick Surveillance Scheme are available from the study authors upon reasonable request.
