# Supplementary material for: Mapping and monitoring tick (Acari, Ixodida) distribution, seasonality, and host associations in the United Kingdom between 2017 and 2020
Source: Med Vet Entomol. 2022 Oct 30;37(1):152–63. doi: 10.1111/mve.12621 (PMC10092223; doi:10.1111/mve.12621)
Supplement: Supplementary file 1 — Table S1: Tick Surveillance Scheme records received 2017–2020, detailing species, host associations [number of records per host species/environment], number of records, number of larvae (L), nymphs (N), adult females (F), adult males (M), and total ticks received. [file MVE-37-152-s002.docx]

Supplementary Table 1: Tick Surveillance Scheme records received 2017-2020, detailing species, host associations [number of records per host species/environment], number of records, number of larvae (L), nymphs (N), adult females (F) and adult males (M) and total ticks received.

| Tick species | Host category | Host species/environment | Records | F | M | N | L | Total ticks |
| --- | --- | --- | --- | --- | --- | --- | --- | --- |
| *Amblyomma americanum** | Human | Attached [2] | **2** | **1** | **0** | **1** | **0** | **2** |
| *Amblyomma hebraeum** | Human | Attached [1] | **1** | **1** | **0** | **0** | **0** | **1** |
| *Amblyomma triguttatum** | Human | Attached [1] | **1** | **0** | **0** | **1** | **0** | **1** |
| *Argas vespertilionis* | Environment | Indoors in properties with bat roosts | **3** | **2** | **0** | **2** | **0** | **4** |
| *Dermacentor auratus** | Human | Attached [2] | **2** | **0** | **0** | **2** | **0** | **2** |
| *Dermacentor marginatus** | Animal | Dog | 1 | 1 | 0 | 0 | 0 | 1 |
|  | Human | Attached [1] | 1 | 0 | 1 | 0 | 0 | 1 |
|  |  |  | **2** | **1** | **1** | **0** | **0** | **2** |
| *Dermacentor reticulatus* | Animal | Dog | 45 | 105 | 72 | 0 | 0 | 177 |
|  | Environment | Indoors | 6 | 13 | 8 | 0 | 0 | 21 |
|  | Human | Found crawling [8], attached [6] | 14 | 9 | 5 | 0 | 0 | 14 |
| History of travel overseas | All hosts | Dog [1 imported] | 3 | 2 | 1 | 0 | 0 | 3 |
|  |  |  | **68** | **129** | **86** | **0** | **0** | **215** |
| *Dermacentor variabilis** | Animal | Dog | **2** | **1** | **1** | **0** | **0** | **2** |
| *Haemaphysalis elliptica** | Animal | Dog [2], cat [1] | **3** | **2** | **1** | **0** | **0** | **3** |
| *Haemaphysalis punctata* | Animal | Horse [38], sheep [4], dog [4], cat [1] | 47 | 134 | 40 | 36 | 18 | 228 |
|  | Human | Found crawling [5], attached [19] | 24 | 17 | 9 | 0 | 0 | 26 |
|  |  |  | **71** | **151** | **49** | **36** | **18** | **254** |
| *Haemaphysalis tibetinensis** | Human | Attached [1] | **1** | **1** | **0** | **0** | **0** | **1** |
| *Hyalomma aegyptium** | Human | Attached [2] | **2** | **0** | **0** | **2** | **0** | **2** |
| *Hyalomma lusitanicum** | Animal | Dog | 1 | 0 | 1 | 0 | 0 | 1 |
|  | Human | Attached [3] | 3 | 2 | 1 | 0 | 0 | 3 |
|  |  |  | **4** | **2** | **2** | **0** | **0** | **4** |
| *Hyalomma marginatum* | Human | Found crawling | **1** | **0** | **1** | **0** | **0** | **1** |
| *Hyalomma rufipes* | Animal | Horse | **1** | **0** | **1** | **0** | **0** | **1** |
| *Hyalomma truncatum** | Human | Attached [1] | **1** | **0** | **1** | **0** | **0** | **1** |
| *Ixodes caledonicus* | Animal | Chough | **1** | **1** | **0** | **0** | **0** | **1** |
| *Ixodes canisuga* | Animal | Fox [22], dog [4], ferret [2], badger [1] | **29** | **60** | **0** | **13** | **20** | **93** |
| *Ixodes frontalis* | Animal | Collared dove [5], house sparrow [5], blackbird [4], greenfinch [2], common wood pigeon [2], blue tit [1], goose [1], kestrel [1], robin [1], starling [1], willow warbler [1], unknown [1] | 25 | 60 | 0 | 3 | 1 | 64 |
|  | Environment | Outdoors [2] | 2 | 2 | 0 | 0 | 0 | 2 |
|  | Human | Attached [5] | 5 | 5 | 0 | 0 | 0 | 5 |
|  |  |  | **32** | **67** | **0** | **3** | **1** | **71** |
| *Ixodes hexagonus* | Animal | Dog [214], cat [163], hedgehog [113], fox [22], ferret [7], badger [2], sheep [2], horse [1], pig [1], polecat [1], unknown [1] | 528 | 2136 | 80 | 1448 | 591 | 4255 |
|  | Environment | Indoors [5], outdoors [6] | 11 | 10 | 4 | 4 | 1 | 19 |
|  | Human | Attached [37], found crawling [3] | 40 | 20 | 2 | 16 | 2 | 40 |
| History of travel overseas | All hosts | Dog [1 imported] | 5 | 4 | 0 | 2 | 2 | 8 |
|  |  |  | **584** | **2170** | **86** | **1470** | **596** | **4322** |
| *Ixodes holocyclus** | Human | Attached [1] | **1** | **0** | **0** | **1** | **0** | **1** |
| *Ixodes ricinus* | Animal | Dog [1065], cat [232], horse [10], deer [11], cow [9], goat [7], sand lizard [6], sheep [6], rabbit [4], squirrel [3], guinea pig [3], hare [3], common lizard [2], pony [2], chicken [1], donkey [1], hedgehog [1], pheasant [1], mute swan[1], siskin [1], pig [1], fox [1], field vole [1], rat [1], grey squirrel [1], unknown [5] | 1379 | 2113 | 417 | 208 | 31 | 2769 |
|  | Environment | Indoors [42], outdoors [11], in vehicle [1] | 54 | 48 | 21 | 31 | 2 | 102 |
|  | Human | Attached [1305], found crawling [40] | 1345 | 279 | 34 | 1121 | 278 | 1641 |
| History of travel overseas | Animal | Dog [18; 10 imported] | 18 | 33 | 0 | 0 | 0 | 33 |
|  | Environment | indoors [3; 1 imported] | 3 | 2 | 0 | 1 | 0 | 3 |
|  | Human | human [39; 18 imported, all attached] | 39 | 17 | 0 | 23 | 1 | 41 |
|  |  |  | **2838** | **2492** | **472** | **1384** | **241** | **4589** |
| *Ixodes scapularis** | Human | Attached [2] | **2** | **1** | **0** | **1** | **0** | **2** |
| *Ixodes trianguliceps* | Animal | Dog | 1 | 1 | 0 | 0 | 0 | 1 |
|  | Environment | Indoors | 1 | 0 | 1 | 0 | 0 | 1 |
|  |  |  | **2** | **1** | **1** | **0** | **0** | **2** |
| *Ixodes ventalloi* | Animal | Cat [5], dog [1] | **6** | **13** | **1** | **0** | **0** | **14** |
| *Ixodes spp.* | Animal | Cat [1], cow [1], dog [1], mouse [1] | 4 | 4 | 0 | 0 | 0 | 4 |
|  | Human | Attached [9] | 9 | 3 | 1 | 2 | 2 | 8 |
|  |  |  | **13** | **7** | **1** | **2** | **2** | **12** |
| *Rhipicephalus appendiculatus** | Human | Attached [1] | **1** | **0** | **0** | **1** | **0** | **1** |
| *Rhipicephalus gertrudae** | Human | Attached [1] | **1** | **0** | **1** | **0** | **0** | **1** |
|  |  |  |  |  |  |  |  |  |
| *Rhipicephalus sanguineus* s.l. | Animal | Dog | 3 | 2 | 0 | 1 | 0 | 3 |
| History of travel overseas | Animal | Dog [34], cat [1] | 35 | 54 | 34 | 316 | 5 | 409 |
|  | Human | Attached [1, imported] | 1 | 1 | 0 | 0 | 0 | 1 |
|  |  |  | **39** | **57** | **35** | **317** | **5** | **413** |
| *Rhipicephalus turanicus** | Animal  Human | Dog  Attached [1] | 4  1  **5** | 3  0  **3** | 1  1  **2** | 1  0  **1** | 0  0  **0** | 5  1  **6** |
| **All species** |  | **All hosts** | **3720** | **5243** | **741** | **3255** | **883** | **10122** |

Latin names for hosts: badger (*Meles meles*)*;* blackbird (*Turdus merula*); blue tit (*Cyanistes caeruleus*); Brown hare (*Lepus europaus*); cat (*Felis catus*); chicken (*Gallus gallus domesticus*); chough (*Pyrrhocorax pyrrhocorax*); collared dove (*Streptopelia decaocto*); common lizard (*Zootoca vivipara*); common wood pigeon (*Columba palumbus*); cow (*Bos taurus*); deer (*Cervus nippon, Capreolus capreolus, Cervus elaphus*); dog (*Canis lupus familiaris*); donkey (Equus asinus); ferret (*Mustela putorius furo*); field vole (*Microtus agrestis);* fox (*Vulpes vulpes*); goat (*Capra aegagrus hircus*); goose (*Anser* sp.); greenfinch (*Chloris chloris*); grey squirrel (*Sciurus carolinensis*); guinea pig (*Cavia porcellus*); hedgehog (*Erinaceus europaeus*); horse (*Equus caballus*); house sparrow (*Passer domesticus*); kestrel (*Falco tinnunculus*); mouse (*Mus/Apodemus* sp.); mute swan (*Cygnus olor*); pig (*Sus scrofa domesticus*); pheasant (*Phasianus colchicus*); polecat (*Mustela putorius*); rabbit (*Oryctolagus cuniculus*); robin (*Erithacus rubecula*); sand lizard (*Lacerta agilis*); sheep (*Ovis aries*); siskin (*Spinus spinus*); starling (*Sturnus vulgaris*); squirrel (*Sciurus vulgaris);* rat (*Rattus* sp.); willow warbler (*Phylloscopus trochilus*)

*imported into the United Kingdom only. Numbers in bold show totals per tick species overall.

Where host category is human only, [ ] specifies the number of records with ticks attached or crawling. Where host is animal and multiple host species are recorded, [ ] shows how many records were associated with each host species.
