# Supplementary material for: Mapping and monitoring tick (Acari, Ixodida) distribution, seasonality, and host associations in the United Kingdom between 2017 and 2020
Source: Med Vet Entomol. 2022 Oct 30;37(1):152–63. doi: 10.1111/mve.12621 (PMC10092223; doi:10.1111/mve.12621)
Supplement: Supplementary file 2 — Table S2: Outputs from the generalized linear mixed effect model (GLMM) explaining the effects month, host, and year on the number of I. ricinus records submitted through the TSS in 2010–2016 Table S3: Outputs from the GLMM explaining the effects month, host, and year on the number of I. ricinus records submitted through the TSS in 2017–2020 [file MVE-37-152-s001.docx]

Supplementary Table 2: Outputs from the generalized linear mixed effect model explaining the effects month, host and year on the number of *I. ricinus* records submitted through the TSS in 2010-2016

|  | Estimate | Std. Error | z-value | p-value | ΔAICc* |
| --- | --- | --- | --- | --- | --- |
| Intercept | 1.80 | 0.12 | 14.57 | <0.001 |  |
| **Host (baseline: Cat)** |  |  |  |  |  |
| Dog | 1.30 | 0.16 | 8.27 | <0.001 |  |
| Human | 1.14 | 0.16 | 7.00 | <0.001 |  |
| Year | 0.64 | 0.05 | 13.20 | <0.001 | 121.5 |
| Month | -0.26 | 0.12 | -2.24 | 0.03 |  |
| Month^2^ | -0.69 | 0.12 | -5.71 | <0.001 |  |
| **Host (baseline: Cat) * month^2^** |  |  |  |  | 11.5 |
| Dog * month | 0.33 | 0.14 | 2.31 | 0.02 |  |
| Dog * month^2^ | -0.29 | 0.15 | -1.97 | 0.05 |  |
| Human * month | 0.30 | 0.16 | 1.82 | 0.07 |  |
| Human * month^2^ | -0.66 | 0.18 | -3.71 | <0.001 |  |

*The ΔAICc refers to the effect of removing the variable in the given row on the AICc of the best model. For example, a ΔAICc of 10 means that the AICc of the model increased by 10 after removing the variable.

Supplementary Table 3: Outputs from the generalized linear mixed effect model explaining the effects month, host and year on the number of *I. ricinus* records submitted through the TSS in 2017-2020

|  | Estimate | Std. Error | z-value | p-value | ΔAICc* |
| --- | --- | --- | --- | --- | --- |
| Intercept | 2.10 | 0.14 | 14.89 | <0.001 |  |
| **Host (baseline: Cat)** |  |  |  |  |  |
| Dog | 1.77 | 0.18 | 9.77 | <0.001 |  |
| Human | 2.14 | 0.18 | 11.78 | <0.001 |  |
| Month | -0.33 | 0.13 | -2.48 | 0.01 |  |
| Month^2^ | -0.57 | 0.14 | -4.01 | <0.001 |  |
| **Host (baseline: Cat) * month^2^** |  |  |  |  | 19.2 |
| Dog * month | 0.16 | 0.16 | 0.97 | 0.33 |  |
| Dog * month^2^ | -0.65 | 0.18 | -3.66 | <0.001 |  |
| Human * month | 0.29 | 0.17 | 1.70 | 0.09 |  |
| Human * month^2^ | -0.98 | 0.19 | -5.23 | <0.001 |  |

*The ΔAICc refers to the effect of removing the variable in the given row on the AICc of the best model. For example, a ΔAICc of 10 means that the AICc of the model increased by 10 after removing the variable.
